# Supplementary material for: Chromothripsis during telomere crisis is independent of NHEJ, and consistent with a replicative origin
Source: Genome Res. 2019 May;29(5):737–49. doi: 10.1101/gr.240705.118 (PMC6499312; doi:10.1101/gr.240705.118)
Supplement: Supplemental Material [file supp_gr.240705.118_Supplemental_file_1.zip › contigs/annotated_contigs/DB92/contig.2.DB92_length_445_mean_cov_10.1123595506.docx]

**DB92_length_445_mean_cov_10.1123595506**

```
AACATTTTAGTTTAGTCTTATTTTAGAGAGGCTCACTTCTAAGGATTTTTTTAATTCAACATGGTTAAAATGTAGACAAATTTAATTTA
 >chrX:12357240-12357381 + E=3e-73
TTGAACCCCAAATCATGTTTTTTGAATGTTCCAAGGCATTTTTCTCATATCA|TCTCATATC|TTGATAACCCTAGTTGGCCTACTAAA
                                                              >chrX:12358326-12358621 -
GGACAAATTGCCTACTTTGACCTCCTTCCCAACTAACTAATCAGTTTCCCCCAAAATAGGAACCAACATTCACTCTTCCTAGGCTGGTT
 E=4e-163
TTTCTCTCTCTTCTCTCATATAAAGCCAGTTTTTCTCTTCTTTATTCTTTGACAATACTCCATAGACCATTTTACACATAACTAAATGT

GCATGCAAATGATTAAACATGTCTAAAAAATGCTAAGTATTGAAACGTTGTTTTCACTTCCACTGGAAAAACTGACATTTATGCAGTGG

TT
```
